# Supplementary material for: The impact of smartphone use on gait in young adults: Cognitive load vs posture of texting
Source: PLoS One. 2020 Oct 12;15(10):e0240118. doi: 10.1371/journal.pone.0240118 (PMC7549775; doi:10.1371/journal.pone.0240118)
Supplement: Attachment — Submitted filename: Response to Reviewers.docx [file pone.0240118.s002.docx]

- **Journal: PLOS ONE**
- **Manuscript Number: PONE-D-20-10060R1**
- **Manuscript Title: The effect of smartphone use on gait in healthy young adults: a randomized, repeated measures, counterbalanced, crossover and single-blind study**
- **Article Type: Research Article**

I would like to thank you for providing the opportunity to revise and resubmit the attached manuscript entitled “The effect of smartphone use on gait in healthy young adults: a randomized, repeated measures, counterbalanced, crossover and single-blind study” for publication in PLOS ONE.

We deeply appreciate the editorial comments on our manuscript which we ignored. We agreed with the points addressed by the Editor and provide our responses to the Editor’ comments as follows:

***Comments from Editor:***

**Journal Requirements**

***1. [Please ensure that your manuscript meets PLOS ONE's style requirements, including those for file naming. The PLOS ONE style templates can be found at***
[***http://www.journals.plos.org/plosone/s/file?id=wjVg/PLOSOne_formatting_sample_main_body.pdf***](http://www.journals.plos.org/plosone/s/file?id=wjVg/PLOSOne_formatting_sample_main_body.pdf) ***and*** [***http://www.journals.plos.org/plosone/s/file?id=ba62/PLOSOne_formatting_sample_title_authors_affiliations.pdf***](http://www.journals.plos.org/plosone/s/file?id=ba62/PLOSOne_formatting_sample_title_authors_affiliations.pdf)***]***

Response:

- According to your suggestion, our manuscript has been modified to fit PLOS ONE's style. Also, file naming has been modified.
- In more detail, in Manuscript, 'Heading font size', 'Figure Citations', and 'File Naming for Figures' have all been changed according to the format of the journal.
- In addition, the attached file name was also modified in accordance with the Submission Guideline.
- If you have another problem, please let me know.

***2. [Please ensure all data are included in the manuscript, as you indicated in the data availability statement.]***

Response:

- Thank you for your careful attention. According to the reviewer's point, 'Data Availability Statement' was added at the end of the Manuscript.

***3. [We note that Figure 1 includes an image of a participant in the study.
As per the PLOS ONE policy (***[***http://journals.plos.org/plosone/s/submission-guidelines#loc-human-subjects-research***](http://journals.plos.org/plosone/s/submission-guidelines#loc-human-subjects-research)***) on papers that include identifying, or potentially identifying, information, the individual(s) or parent(s)/guardian(s) must be informed of the terms of the PLOS open-access (CC-BY) license and provide specific permission for publication of these details under the terms of this license. Please download the Consent Form for Publication in a PLOS Journal (***[***http://journals.plos.org/plosone/s/file?id=8ce6/plos-consent-form-english.pdf***](http://journals.plos.org/plosone/s/file?id=8ce6/plos-consent-form-english.pdf)***). The signed consent form should not be submitted with the manuscript, but should be securely filed in the individual's case notes. Please amend the methods section and ethics statement of the manuscript to explicitly state that the patient/participant has provided consent for publication: “******The individual in this manuscript has given written informed consent (as outlined in PLOS consent form) to publish these case details”.***

***If you are unable to obtain consent from the subject of the photograph, you will need to remove the figure and any other textual identifying information or case descriptions for this individual.]***

Response:

- Thank you for your detailed point. We received a consent from participant for the use of the image in the manuscript, and added relevant information at the Methods section as below:
- 1^st^ ver.: “This study was approved by the Institutional Review Board of the Gachon University (1044396-201907-HR-126-01), and enrolled in the Clinical Research Information Service that complies with the World Health Organization International Clinical Trials Registry Platform (WHO-ICTRP) (registration number: KCT0004875). All procedures were done in compliance with the Declaration of Helsinki.”
- 2^nd^ ver. (Line 74-78): “This study was approved by the Institutional Review Board of the Gachon University (1044396-201907-HR-126-01), and enrolled in the Clinical Research Information Service that complies with the World Health Organization International Clinical Trials Registry Platform (WHO-ICTRP) (registration number: KCT0004875). All procedures were done in compliance with the Declaration of Helsinki. The individual in this manuscript has given written informed consent (as outlined in PLOS consent form) to publish these case details.”

**Additional Editor Comments**

***1. [The study is presented as a clinical trial, but there is only 1 group and all subjects participated in all aspects, please clarify.]***

Response:

- First of all, we thank you for the Editor’s careful attention. As you know, our study was designed as a clinical trial, among which is a screening trial. As everyone knows, it is not clear to date that smartphone use directly causes specific accidents or motor dysfunction in the users. This study is a screening step to identify the factors that smartphone use can cause in this event or disease.
- More precisely, the design of this study is a single-arm clinical trial. The following site is attached as a reference for the research.
- <https://clinicaltrials.gov/ct2/show/NCT02018042>
- We described this in line 81 of the Manuscript as 'This study was conducted with a single group repeated measure design'. Please point out if there is a problem or correction in this expression.
- As you thought, all subjects participated in all measurements. This is described in the Manuscript Participants section as follow:
- Line 78-79: “All subjects in this study participated in all aspects of measurements.”

***2. [It is not clear how this study evaluated walking safety. These claims appear to be over-stated or unsubstantiated, please clarify.]***

Response:

- Thank you for your detailed point. As per the Editor's comments, we modified the expressions used for [walking safety] to the original purpose as follows;
- 1^st^ ver.: “This study demonstrates that smartphone use has a negative effect on pedestrian safety and walking ability.”
- 2^nd^ ver. (Line 40): “This study demonstrates that smartphone use has a negative effect on ~~pedestrian safety and~~ walking ability.
- 1^st^ ver.: “This study was intended to investigate the effects of smartphone use and postural change on the stability and walking ability of pedestrians by comparing the effects of smartphone use on spatiotemporal gait parameters in healthy young people, and changes in parameters according to smartphone use posture and task.”
- 2^nd^ ver. (Line 57-59): “The purpose of this study was investigate the effects of smartphone use and postural change on ~~the stability and~~ the walking ability of pedestrians by comparing the effects of smartphone use on spatiotemporal gait parameters in healthy young people, and changes in parameters according to smartphone use posture and task.”
- 1^st^ ver.: “In general, gait speed is used as an indicator for vital signs or to predict pedestrian safety.”
- 2^nd^ ver. (Line 191): “Many gait parameters are used as an indicator for gait ability or dysfunction.”

***3. [Lines 45-48 are clumsy, consider removing or significantly re-phrasing since they read as opinions. 39% market penetration is not "essential... [for] daily life of modern people"]***

Response:

- For the part you pointed out, the clumsy content and the statistical part were corrected as follows:
- 1^st^ ver.: “Smartphones are one of the most essential items in the daily life of modern people. A smartphone is a smart device that provides various functions such as TV, games, the Internet, e-commerce, and documentation, as well as text messaging and calling. In addition, it has spread rapidly thanks to its advantage of being able to be used easily regardless of time and place. In 2018, the global smartphone penetration rate reached 39.5%, and Korea ranked 11th in the world. It has been reported that more than 34 million people are using smartphones in Korea [1, 2]. Thus, smartphone use has become a part of our life, providing convenience and influencing users' daily lives in various ways, but conversely, smartphones continuously raise the issue of dysfunction.”
- 2^nd^ ver. (Line 43-47): “Smartphones are one of the most essential items in the daily life of modern people. In 2018, the smartphone penetration rate exceeded 70% in more than 9 countries, including the United States and the United Kingdom, and more than 250 million people in the United States are using smartphones. Korea's smartphone penetration rate is 11th in the world, and more than 34 million people are using it [1, 2]. Along with the rapid spread of smartphones, the problems caused by the use of smartphones are also increasing.”

***4. [Paragraph 2 on phone use during driving should be condensed to a single sentence and integrated into the following paragraph.]***

Response:

- Thank you for your detailed recommendations. We revised the introduction as follows according to the method you suggested;
- 1^st^ ver.: “Traffic accidents caused by smartphone use have recently become a social issue as one of the negative aspects of smartphones. Smartphone use reduces a pedestrian’s situational awareness and distracts their attention and working memory. It also distracts drivers, leading to negative effects on visual information on the road, motor control and response, and the high-level cognitive processing of drivers. Thus, smartphone use on the road leads to increased accident rates for both drivers and pedestrians.

In addition to the degraded cognitive function caused by smartphone use, some studies have reported that smartphone use deforms posture and change gait patterns. Schabrun et al. [12] reported that smartphone use affects gait by reducing the trunk movement of pedestrians, and Jeon et al. [13] reported that there are differences in gait patterns according to the difficulty of tasks using smartphones. Many factors are involved in gait, but many studies only focus on the analysis of changes in gait speed, and do not analyze other parameters related to gait. Therefore, analysis of various spatiotemporal gait parameters is required to clearly understand the effect of smartphone use on gait.”

- 2^nd^ ver. (Line 48-56): “~~Traffic accidents caused by smartphone use have recently become a social issue as one of the negative aspects of smartphones. Smartphone use reduces a pedestrian’s situational awareness and distracts their attention and working memory. It also distracts drivers, leading to negative effects on visual information on the road, motor control and response, and the high-level cognitive processing of drivers. Thus, smartphone use on the road leads to increased accident rates for both drivers and pedestrians.~~

The use of smartphones while walking reduces the situational awareness and distracts attention and work memory, negatively affecting visual information on the road, motor control and. In addition to the degraded cognitive function caused by smartphone use, some studies have reported that smartphone use deforms posture and change gait patterns. Schabrun et al. reported that smartphone use affects gait by reducing the trunk movement of pedestrians, and Jeon et al. reported that there are differences in gait patterns according to the difficulty of tasks using smartphones. Many factors are involved in gait, but many studies only focus on the analysis of changes in gait speed, and do not analyze other parameters related to gait. Therefore, analysis of various spatiotemporal gait parameters is required to clearly understand the effect of smartphone use on gait.”

***5. [Line 79, please clarify whether the screening happened prior to the informed consent.]***

Response:

- Of course, we went through all the procedures in compliance with research ethics. However, the explanation of lack of expression was corrected as follows:
- 1^st^ ver.: “A total of 62 people volunteered for this study, and those who did not meet the inclusion criteria after the research physiotherapist’s screening were excluded from the study. Finally, 42 healthy young adults participated in this study. All participants received a full explanation about this study before signing a consent form.”
- 2^nd^ ver. (Line 70-74): “The 62 participants who indicated their willingness to participate in the study received a full explanation about this study before signing a consent form. Volunteers performed a screening after signing the consent form and those who did not meet the inclusion criteria after the research physiotherapist’s screening were excluded from the study. Finally, 42 healthy young adults participated in this study.”

***6. [Line 90 is unclear]***

Response:

- The blinded method pointed out was further explained as follows:
- 1^st^ ver.: “All measurements were taken under blinded conditions.”
- 2^nd^ ver. (Line 86): “All measurements and analyzes were performed by researchers blinded to the purpose and method of the study.”

***7. [Line 105, it is not clear what they were instructed to type.]***

Response:

- Thank you for pointed-out about the lack of explanation of the intervention. We have changed the description of this part as follows:
- 1^st^ ver.: “They were instructed to type in ascending or descending order each time, starting with a random number from 0 to 20, according to the evaluator's instructions.”
- 2^nd^ ver. (Line 100-103): “They were instructed to enter in ascending or descending direction, starting with a random number between 0 and 20. The ascending or descending direction and random number were determined using the website randomization.com (http://www.randomization.com).”

***8. [Line 146, please clarify if these subjects were "removed" by the investigators rather than "dropped out" since dropping out indicates a voluntary choice by the subject.]***

Response:

- Thanks for your detailed recommendations. We substituted ‘dropped out’ to ‘removed’ as per your comments.
- 1^st^ ver.: “When a contaminated factor that might affect spatiotemporal gait parameters occurred (complaining about discomfort due to barefoot gait (n = 5), abnormal data due to apparatus error (n = 1)), it was considered to dropped out.”
- 2^nd^ ver. (Line 144-146): “When a contaminated factor that might affect spatiotemporal gait parameters occurred (complaining about discomfort due to barefoot gait (n = 5), abnormal data due to apparatus error (n = 1)), it was considered to removed.”

***9. [It is not clear from the design or methods how the present study demonstrates a change in cognitive function or posture, this seems like a missed opportunity. There are several areas with claims regarding cognitive distraction or load and it is not clear how that was evaluated, this must be addressed.]***

Response:

- Fortunately, we have already considered the part presented by the Editor, and we have further described this in the Method section.
- The rationale for the cognitive distraction or load we described was based on previous studies that proved that a dual task require a demand for cognition rather than a single task. In addition, by comparing a dual task and a single task, the method of identifying the effects on a primary task (common element of two tasks) and a secondary task (additional element) is a commonly used method. Therefore, we thought that the editor's proposal for 'smartphone use during gait increased cognitive distraction or load' was identified through various postural conditions of smartphone use in this research.
- This study compared the following three postures: (1) walking without a smartphone use, (2) smartphone use during walking (cognitive dual task), and (3) posture without smartphone use (single task).
- Through these, the study identified two things as follow:
  - (1) Effects of secondary task following smartphone use on user's cognition
  - (2) Effects of various postures using a smartphone (between each single task without cognition task) on walking
- Frank, James S., and Marie Earl. "Coordination of posture and movement." *Physical therapy* 70.12 (1990): 855-863.
- Horak, Fay B., H. C. Diener, and L. M. Nashner. "Influence of central set on human postural responses." *Journal of neurophysiology* 62.4 (1989): 841-853.
- Horak, Fay B., and Lewis M. Nashner. "Central programming of postural movements: adaptation to altered support-surface configurations." *Journal of neurophysiology* 55.6 (1986): 1369-1381.
- Quant, Sylvia, et al. "The effect of a concurrent cognitive task on cortical potentials evoked by unpredictable balance perturbations." *BMC neuroscience* 5.1 (2004): 18.
- Quant, Sylvia, Brian E. Maki, and William E. McIlroy. "The association between later cortical potentials and later phases of postural reactions evoked by perturbations to upright stance." *Neuroscience letters* 381.3 (2005): 269-274.
- Ansai, Juliana Hotta, Thais Rabiatti Aurichio, and José Rubens Rebelatto. "Relationship between dual task walking, cognition, and depression in oldest old people." *International psychogeriatrics* 28.1 (2016): 31-38.
- Cocchini, Gianna, et al. "Dual task effects of walking when talking in Alzheimer's disease." *Revue Neurologique* 160.1 (2004): 74-80.
- 1^st^ ver.: “In the BS condition, they were instructed to use a smartphone with both hands, and position the smartphone with a comfortable posture and at a comfortable hand height for typing. In the DS condition, they were instructed to use the smartphone with their dominant hand and place the smartphone in a comfortable position for texting. The other arm was ordered to swing or lower naturally. To ensure that the PS condition is in the same posture as the BS condition, they were instructed to type numbers for 5 seconds while standing on the starting line before starting to walk and then turn off the screen and walk while maintaining the posture.”
- 2^nd^ ver. (Line 104-112): “In the BS condition, they were instructed to use a smartphone with both hands, and position the smartphone with a comfortable posture and at a comfortable hand height for typing. In the DS condition, they were instructed to use the smartphone with their dominant hand and place the smartphone in a comfortable position for texting. The other arm was ordered to swing or lower naturally. To ensure that the PS condition is in the same posture as the BS condition, they were instructed to type numbers for 5 seconds while standing on the starting line before starting to walk and then turn off the screen and walk while maintaining the posture. To exclude the cognition burden, PS condition is designed. PS condition is to reproduce the same attitude as using a smartphone without using the smartphone. BS and DS condition are cognitive dual tasks where both walking and texting tasks are performed simultaneously, and PS condition is a single task that walks without texting tasks.”

***10. [Gait speed and balance are not equivalent. See recent work by John Jeka and others in the field of postural control during walking]***

Response:

- Thank you very much for your valuable point and we agree on this. As per the your point, we revised the content expressed in the Discussion section as follow:
- 1^st^ ver.: “In general, gait speed is used as an indicator for vital signs or to predict pedestrian safety [18]. Verghese et al. [19] reported that fall risk increases by 7% as gait speed decreases by 10 cm / s. The results of our study show that gait speed (-27.94 cm / s) decreases while walking and texting with both hands compared to normal walking, which indicates that smartphone use while walking negatively affects a pedestrian’s ability to balance. In particular, gait speed (-13.2cm / s) decreases even while walking in PS condition, which indicates that postural deformation due to smartphone use may have an effect on increasing the fall risk of pedestrians.”
- 2^nd^ ver. (Line 191-197): “Many gait parameters are used as an indicator for gait ability or dysfunction [18]. Verghese et al. [19] who analyzed gait parameters and gait ability, reported that gait speed and fall risk were related, and suggested variables such as swing time, double-support phase, and stride length as gait parameters that can predict fall. The results of our study show that preferred gait speed (-27.94 cm / s) decreases while walking and texting with both hands compared to normal walking. In particular, preferred gait speed (-13.2cm / s) decreases even while walking in PS condition. These results indicates that changes in postural as well as cognition caused by the use of smartphones can negatively affect the gait ability of pedestrians. ”

***11. [The manuscript is concurrently being evaluated for methodology and statistical design, but the above issues need to be addressed with a primary revision responded to in a point by point manner before this manuscript can be reconsidered for peer review.]***

Response:

- All authors checked the contents of all comments received from the editor, and this author wrote a response letter with the consent of all authors.
- In particular, we checked whether there were any problems with Methodology and Statistical design, and finally modified the manuscript.
- We hope that the modified and submitted manuscript will be satisfied with the Editorial office. Please tell us if you have any other problems. I will respond actively and also correct it.

We hope that the revisions we have made are satisfactory. Please inform us if there is anything else that can be done to improve the manuscript.

Thank you for your consideration.

Sincerely,

**Hwi-young** **Cho**, P.T., Ph.D.

Associate Professor

Department of Physical Therapy, Gachon University College of Health Science

191 Hambangmoe-ro, Yeonsu-gu, Incheon, 21936, Republic of Korea

Email address: [hwiyoung@gachon.ac.kr](mailto:hwiyoung@gachon.ac.kr)

Tel: +82-32-820-4560; Fax: +82-32-820-4420
